# Response and recovery kinetics of a solid tumour after irradiation.

**DOI:** 10.1038/bjc.1980.283

**Published:** 1980-10

**Authors:** R. Rowley, H. A. Hopkins, W. L. Betsill, E. R. Ritenour, W. B. Looney

## Abstract

**Images:**


*Br. J. Cancer* (1980) **42**, 586

### RESPONSE AND RECOVERY KINETICS OF A SOLID TUMOUR AFTER IRRADIATION

R. ROWLEY*, H. A. HOPKINS*, W. L. BETSILL, JR.†, E. R. RITENOUR* AND W. B. LOONEY*

*From the *Division of Radiobiology and Biophysics, University of Virginia School of Medicine, and †Department of Pathology, University of Virginia Medical Center, Charlottesville, Virginia* 22908

Received 2 January 1980 Accepted 23 June 1980

**Summary.—The effects of local tumour radiation over the dose range 7·5–30 Gy on the growth and cell kinetics of rat hepatoma H-4-II-E have been investigated. A plot of growth delays against log surviving fraction was linear below a fraction of 0·03, but failed to extrapolate to the origin. Following a single dose of 15 Gy to the tumour, DNA-precursor incorporation, labelling and mitotic indices were depressed for 7 days. Tumour cellularity, measured as DNA/g tumour, was reduced and the rate of increase of total clonogenic cells slower than after complete tumour recovery. From Day 7 to Day 9 all indices of proliferation recovered to about control levels, clonogenic cell numbers increased more rapidly and tumour cellularity was restored. Repopulation of the tumour therefore appeared to take place mainly after Day 7. Incorporation of [$^3$H]-TdR into tumour DNA reached twice the control values on Day 9. The rate of tumour growth accelerated after the initial decrease, and maximum tumour growth rate was also twice the control values on Day 13. Accelerated growth rates in irradiated tumours, above those of control tumours, occurred 10–16 days after treatment. The effectiveness of sequential therapy may therefore be improved if given during this period of accelerated tumour growth.**

THE KINETICS of tumour cell population recovery following X-irradiation are not well known, thus, the factors governing the timing of tumour recovery are even less well understood. This lack of knowledge results in part from the following factors: too few studies have documented both tumour volume and tumour cell kinetic changes simultaneously (Barendsen & Broerse, 1969 and Hermens, 1973, are notable exceptions), the results of such investigations may be confused by repair phenomena (Twentyman & Bleehen, 1975) or host factors (Janik & Steel, 1972), and the validity of assays necessary for determining tumour cell population changes, in particular the clonogenic assay, is uncertain (McNally, 1973).

In an attempt to overcome some of these difficulties, we have examined the effects of single doses of radiation upon a hepatoma, grown s.c., combining assays for volume, cell kinetic, cell survival and biochemical changes. The tumour line, H-4-II-E, is derived from the Reuber hepatoma H-35 (Evans & Kovacs, 1977). It readily metastasizes to the lungs and regional lymph nodes and is resistant to radiation and three types of chemotherapeutic agent (adriamycin, cyclophosphamide and 5-fluorouracil). H-4-II-E is thus a realistic tumour model.

### MATERIALS AND METHODS

### *Animals and tumours*

Hepatoma H-4-II-E cells, obtained originally from Dr V. R. Potter, University of Wisconsin, were maintained *in vitro* in Swim's 77 medium with 25% serum (20% horse, 5% foetal bovine, GIBCO, New York). Cells were passed weekly on attaining confluence. *In vitro* growth characteristics are detailed in Kovacs *et al.* (1977).

S.c. inoculation of $2 \times 10^6$ log-phase cells in 0·1 ml serum-free medium into the flank of male ACI rats (Laboratory Supply Company, Indianapolis, IN) produced palpable tumours in 7 to 9 days. Rat weight at inoculation was 120–140 g. Animals were caged individually in an air-conditioned room, lighted from 08:00 to 20:00, with rat chow (Charles River Laboratories, Wilmington, MA) and water *ad libitum.*

### *Irradiation*

X-rays were produced by a General Electric Maximar III 250 (250 kV, 15 mA; filtered with 0·25mm Cu and 1·0mm Al). Before irradiation, animals were anaesthetized with ether and placed in a lead-shielded box through which the tumour protruded. The midpoint of the tumour was ~6 cm from the X-ray-tube target and received the doses indicated, while the animal body received 0·5% of that dose. X-ray output was routinely calibrated with a Victoreen R-meter. Radiation doses are given here as the machine output in grays (Gy). Dose rate was 2·6 Gy/min.

### *Tumour assays*

*Volume.*—Tumour volumes were calculated from the length, width and height, on the assumption that tumours are hemi-ellipsoids, in which volume = 1/2 [4/3 $\pi$ L/2. W/2.H], which reduces to LWH/2. Measurements were made daily. Treatments were given when volumes first exceeded 200 $mm^3$ for volume regrowth experiments, or when in excess of 700 $mm^3$ for clonogenicity and biochemical assays. Volume responses to radiation at these 2 tumour sizes were similar. Tumour-volume growth curves were fitted by the method of least squares with a polynomial of degree 3–6.

Tumour volumes are normalized to the volume on day of treatment and plotted on a semilog scale for each animal. The result is a series of graphs of ln $(V_t/V_0)$ *vs* time where $V_t$ is the tumour volume at time *t* and $V_0$ is the volume at treatment. These tumour-volume growth curves are then fitted by the method of least squares with a polynomial of degree N the functional form of which is

$$\ln\left(\frac{V_t}{V_0}\right) = a_0 + a_1t + a_2t^2 + \ldots a_Nt^N$$

where N values of up to 6 are necessary for a proper fit. Instantaneous tumour growth rate at any time can then be determined by taking the first derivative of the polynomial with respect to time (Looney *et al.*, 1980*b*).

Growth delays were determined as the difference between the time for treated and control tumours to grow from the volume at treatment ($V_0$) to $8 \times V_0$. Time to reach the endpoint volume was calculated for each tumour and the results averaged. Standard errors for growth delay were calculated by the formula $\Delta D = \sqrt{\Delta C^2 + \Delta T^2}$ where $\Delta D$ is the standard error for growth delay, $\Delta C$ = the standard error of the mean time for controls to reach the endpoint volume, and $\Delta T$ = the standard error of the mean time for treated tumours to reach endpoint volume.

*Clonogenicity.*—Tumours were resected from rats killed with ether, weighed and minced with scalpels. The tumour mince was suspended in 37°C buffered trypsin (activity 1:250, DIFCO) at 1 g/30 ml. The mix was stirred for 15 min, filtered through gauze, and diluted with an equal volume of serum medium. Cell numbers were counted by haemacytometer using Crystal Violet stain. Cells were plated into 10cm-diameter plastic Petri dishes. The number of clones containing more than 50 cells was scored after 10 days' incubation, and the proportion of clone-forming cells in treated tumour-cell suspensions expressed as a fraction of that for untreated control tumours.

*DNA content and specific activity.*—One hour before being killed rats were given 50 $\mu$Ci i.p. of $^3$H-(methyl)-thymidine (sp. act. 3 Ci/mM). DNA was extracted from a sample of tumour by procedures described by Hopkins *et al.* (1976), and DNA content/g tumour measured according to Burton (1956). Calf thymus DNA was the standard. Radioactivity in the nucleic acid extracts was measured on a Beckman liquid-scintillation spectrophotometer with external standardization.

*Light microscopy of tissue sections.*—Tumour tissue specimens were fixed in neutral formalin, embedded in paraffin, sectioned and stained by the Feulgen reaction. Slides were dipped in Kodak NTB 2 photographic emulsion for autoradiography, exposed for 3–5 weeks, and developed in Kodak D-19. The percentage of nuclei bearing $>3$ silver grains was scored from random traverses of the section.

Histological evaluation was performed on haematoxylin–eosin stained slides of tumour portions 0·6–1·6 cm in diameter. Only those areas of tumour which appeared morphologically viable were evaluated (*i.e.*, areas with cytoplasmic haematoxylinophilia). Veins measuring 50–100 $\mu$m were used as focal points and all surrounding perivascular tissue within a 1mm radius was examined. Two foci per tumour were evaluated for identification of mitoses. Only obvious mitoses were counted (*e.g.*, metaphase, anaphase, and telophase stages with clearly identifiable chromosomes) (Fig 11A). No examined field contained necrotic tissue. Forty-eight rat tumours were examined histologically.

All slides were evaluated for degree of necrosis, amount of haemorrhage, degree of capsular lymphocytic infiltrates and lymphocytic infiltration of tumour. Nuclear diameters, as well as total cell diameters, were measured with a Zeiss Kpl-W10X ocular micrometer.

Fig. 1A.—Changes in tumour volume after X-irradiation. Each point is the mean for 10 tumours. Vertical bars represent s.e.

Fig. 1B.—Changes in tumour growth rate after 15Gy. ●, mean of the instantaneous growth rates for 8 H-4-II-E tumours on the indicated day after treatment; ○, controls; bars, ± s.e. Length of the period of accelerated growth rate (8·3 days) was determined as the full width at half maximum peak height.

### RESULTS

An effect upon tumour growth was seen by Day 2 after irradiation at all doses (Fig. 1A). Below 15 Gy growth was slowed; exposures of 20 or 30 Gy caused regression. In all instances the treated-tumour growth rates ultimately returned to control levels. Growth rate fell below the control level the first week after 15 Gy. It then accelerated and growth rates comparable to controls were achieved by Day 10 (Fig. 1B). Maximum tumour growth rates after radiation occurred on Day 13.

The log surviving fraction of cells irradiated *in vivo* and immediately assayed for cloning ability *in vitro* decreased exponentially with dose ($D_0 = 4{\cdot}11$ Gy) and without a significant shoulder ($n = 1{\cdot}31$). The plating efficiency (PE) of controls in this experiment was 0·26 (Fig. 2). The relationship between log surviving fraction and growth delay at

FIG. 2.—*In vitro* cloning ability according to tumour dose of X-irradiation *in situ*. Each point is the mean surviving fraction of 3 tumours, relative to the control (1·0). Vertical bars represent ±s.e.

FIG. 3.—Tumour growth delays (mean of 4 experiments) plotted against surviving fraction assayed *in vitro* 1 h after X-irradiation *in vivo*.

equivalent doses is biphasic (Fig. 3). Below 0·03 surviving fraction (15 Gy) the points fit a straight line with a correlation coefficient of 0·967. This curve diverges from the theoretical curve (broken line) that denotes the expected time for a population of tumour cells with a doubling time of 49·2 h (the doubling time of the untreated tumour; Evans & Kovacs, 1977) to return to its pretreatment size.

Clonogenic assays made at intervals after 15 Gy X-rays (Fig. 4) showed that the clonogenic fraction increased exponentially from 0·03 on Day 0 to 1·0 on Day 9 or 10 (control PE = 0·32). This assay requires the enzymic dissociation of the tumour to a single-cell suspension. The cell yield (cells/g untreated tumour) (Fig. 5) was $2{\cdot}5 \times 10^7$ (excluding lymphocytes but not other host cells) and did not show a dependent variation with tumour weight over the range used in these experiments. Yields were reduced from Day 1 to

FIG. 4.—Changes in the *in vitro* clonogenic fraction of H-4-II-E tumours at intervals after 15Gy X-rays. Each point is the mean for 3 tumours when s.e. bars are included, or 2 when absent. The 2 straight-line portions of the curve were fitted separately by regression analysis.

FIG. 5.—Cell yields per tumour dissociated for clonogenic assay, at intervals after irradiating the tumour with 15Gy X-rays. The solid point (●) is the mean for 10 untreated tumours. Open symbols (○) represent the means for 3 treated tumours (the first is 1 h after irradiation). Vertical lines indicate ±s.e. The curve was fitted by eye.

FIG. 6.—Total numbers of clonogenic cells after 15Gy X-rays. Values were determined per tumour. 3 tumours were sampled per point where s.e. bars are included, 2 where absent.

Day 7 after 15 Gy. Total clonogenic cells per tumour (the product of tumour weight at resection, cell yield and clonogenic fraction for individual tumours) increased in 3 phases after irradiation (Fig. 6): Days 0–7 cell numbers increased 10-fold (doubling time, $T_d = 2{\cdot}3$ days), Days 7–9 cell numbers increased 20-fold ($T_d = 0{\cdot}46$ days or 11·2 h) and Day 9 onwards $T_d = 4{\cdot}5$ days. Evans & Kovacs (1977) reported, for

FIG. 7.—Changes in DNA content of H-4-II-E tumours (mg DNA/g tumour) after 15Gy X-rays. Each point is the mean of 3 tumours where there is an s.e. bar, 2 where no bar. ●, untreated; ○, 15 Gy.

FIG. 8.—DNA labelling with time from irradiation. Tumours were sampled 1 h after [$^3$H]-TdR administration to the animal. Points are means of 3 tumours ± s.e. ●, untreated; ○, 15 Gy.

FIG. 9.—Changes in the percentage of labelled cells in tumours sampled at intervals after irradiation. Tritiated thymidine was administered to the animal 1 h before killing. ●, untreated; ○, 15 Gy.

untreated tumours, volume $T_d$ of 49·2 h and *in vivo* cell cycle time of 36 h.

DNA/mg of untreated tumour increased with tumour volume (Fig. 7), possibly because larger tumours contained less blood. After 15 Gy, DNA/mg dropped to a nadir on Day 7 and then recovered to above controls. The specific activity of [$^3$H]-TdR in these DNA samples showed similar kinetics, the recovery phase peaking at twice the control activity (Fig. 8).

Labelling and mitotic indices (Fig. 9 and 10 respectively) initially depressed by irradiation (15 Gy) also recovered at Day 7. Mitoses were always more numer-

FIG. 10.—Changes in the number of mitoses per unit area of sections from tumours sampled at intervals after irradiation. ●, untreated; ○ 15 Gy.

TABLE.—*Percentages of abnormal mitoses and nuclear diameters at intervals after 15 Gy X-rays to the tumour* in situ

| Days after 15 Gy | % Abnormal mitoses ± s.e. | Mean nuclear diameter (μm) |
|---|---|---|
| 1 | 47 ± 3·3 | 15·1 |
| 2 | 33 ± 16·7 | |
| 3 | 83 | |
| 4 | 80 | |
| 7 | 30 ± 15·3 | 17·0 |
| 11 | 20 ± 5·8 | 13·7 |

ous in the perivascular tissue, and decreased in concentration with increasing distance from the vein. The proportion of abnormal mitoses at intervals from irradiation (Table) decreased on Days 7 and 11; however, this was chiefly the result of dilution by an increase in the proportion of normal mitoses at this time.

Little variation in nuclear diameter was evident from comparison of means in tumours sampled at 1, 7 or 11 days after 15 Gy; however, cells showed marked nuclear pleomorphism on Day 7 compared with Day 1, and this situation was largely reversed by Day 11 (Fig. 11A and B).

The degree of necrosis in both treated and untreated tumours progressed with increasing time from Day 0. The degree of haemorrhage present appeared to have no correlation with treatment or time of evaluation. During Day 3 after treatment, lymphocytes and plasma cells accumulated in the capsule surrounding the tumour and superficial parenchymal infiltration of the tumour by lymphocytes was noted. On Day 5 the lymphocytic infiltrate became more prominent, penetrating deeply into the parenchyma *via* small lymphatic channels, and was associated with focal cell necrosis. The lymphocytic infiltrate became less prominent on Day 9, and by Day 11 only scattered lymphocytes were noted in the parenchyma. Capsular lymphocytes and plasma cells were in evidence throughout the duration of this experiment (up to Day 17).

### DISCUSSION

Both the labelling and mitotic indices show that the rate of cell proliferation in

(A) (B)

Fig. 11.—Sections of irradiated tumours (15 Gy) H&E × 180. (A) Day 1—relative monorphism. (B) Day 7—increased pleomorphism.

tumour H-4-II-E was depressed for 7 days after 15 Gy X-rays. The rate of clonogenic cell proliferation was further depressed, since a large proportion of the mitotic figures scored over Days 0–7 were abnormal and most probably represented doomed cells (Table). After Day 7 the rates of cell proliferation increased rapidly to control levels, coinciding with increases in [$^3$H]-TdR incorporation and DNA concentration (a measure of tumour cellularity), with the reinitiation of volume growth at control rates and with the fastest rate of increase in clonogenic cell numbers. These data indicate that repopulation of the tumour with clonogenic cells occurred mainly after Day 7 following 15 Gy of X-rays.

The rate of increase in clonogenic cell numbers (Fig. 6) suggests that cell proliferation 7 days after 15 Gy is faster than in untreated tumours. These data were obtained by sequential sampling of individual tumours from an irradiated population, and thus give a valid indication of post-irradiation changes only if tumour sizes at treatment were similar. For the 36 tumours used in this experiment, maximum and minimum volumes at Day 0 were 1095 and 630 mm$^3$ respectively, and the mean ± s.d. was 824·2 ± 21·2 mm$^3$. The validity of the clonogenic assay itself is of course questionable, and this is referred to later.

The rate of [$^3$H]-TdR incorporation into DNA after irradiation (Fig. 8) is on Day 9 twice the value in age-equivalent controls and also suggests rapid cell proliferation at this time, presumably by a reduction in the length of the S phase. Enhanced incorporation may also result from the induction of partial synchrony.

The accelerated growth rate (Fig. 1B) parallels the increase in specific activity of tumour DNA, but the time sequence is delayed 3–4 days. Maximum labelling of tumour DNA on Day 9 precedes the maximum rate of tumour growth by 4 days.

Tumour DNA labelling and growth rates are about twice the control values at the time of their respective maxima. Studies in patients with lung cancer have shown that an accelerated increase in tumour volume occurs 10–35 days after a reduction in tumour volume following single and fractionated radiotherapy. Studies on metastatic lung tumours from a variety of primary cancers in the dog, on artificially induced metastases in mice, and primary rhabdomyosarcoma in the rat, showed a similarly accelerated increase in tumour volume after a reduction following treatment (Van Peperzeel, 1972; Hermans & Barendsen, 1978). Parallel studies in another rat hepatoma line, 3924A, with different growth, morphological and metastatic characteristics have also shown accelerated tumour growth after both chemotherapy (*i.e.* cyclophosphamide) and radiotherapy (Looney *et al.*, 1980*a*).

Three other reports give evidence of delays (other than mitotic delay) in the proliferation of surviving cells after irradiation. In the R-1 rhabdomyosarcoma, 20 Gy of X-rays reduced the clonogenic fraction to 0·0027 and appeared to delay proliferation of surviving cells for 4 days. Six Gy of 15MeV neutrons reduced the clonogenic fraction to 0·007 but delayed surviving cell proliferation for 6 days, producing an RBE for neutron-induced growth delay of 3·3 but for cell killing 2·8. Cell proliferation, once initiated, was at a rate exceeding that in untreated tumours (Barendsen & Broerse, 1969). In the mouse carcinoma NT, Denekamp & Harris (1976) used X-rays in a 2-dose assay for tumour repopulation. Following 15 Gy an increase in viable cells numbers was not detected for 5 days. Finally, Szczepanski & Trott (1975) monitored repopulation of the C3H mouse adenocarcinoma 284 using [$^3$H]-TdR labelling. After 12 Gy of 300kV X-rays, tumours regressed rapidly, but for 4 days showed little change in growth fraction or cell-cycle length. On Days 4–5 there was a transient and rapid increase in the growth fraction, exceeding that of controls, and apparently due to the triggering of a cohort of quiescent (non-cycling) cells into proliferation. The interval between irradiation and the increase in growth fraction was proportional to dose, and suggested a "dose dependent proliferative delay for resting tumour cells forced to recycle". The authors rejected the possibility that reoxygenation was the factor initiating cell proliferation, since earlier conditions appeared sufficient to support proliferation.

From these reports and our own data it is clear that tumour growth delay cannot simply depend on the fraction of tumour cells that have retained the capacity for unlimited proliferation. Neither then can we expect a linear relationship between growth delay and log surviving fraction. In fact, in the instances (of which we are aware) where this has been determined following irradiation (here; McNally, 1973; Stephens & Steel, 1980), a linear relationship was not observed.

McNally measured the surviving fractions, assayed *in vitro* and the corresponding growth-delays for tumour $RIB_5C$ irradiated under 3 levels of oxygenation: euoxic (animals breathing air), hyperoxic (animals breathing $O_2$) and hypoxic (tumours clamped). Under euoxic conditions of irradiation the increase in delay with decrease in log surviving fraction was less rapid above a surviving fraction of $10^{-1}$ than below. Under hyperoxic or hypoxic conditions delay increased linearly over the whole range, diverging from the curve for euoxic irradiations below a surviving fraction of $10^{-1}$. Thus, for a reduction in surviving fraction to less than $10^{-1}$, greatest growth delay was measured in tumours of air-breathing animals. McNally was unable to ascribe these differences in OER for cell killing and tumour growth delay to an *in vivo* biological mechanism, but suggested instead that there were an artefact of the clonogenic assay. This possibility cannot be eliminated; however, the existence of a "dose dependent proliferative delay for resting tumour cells forced to recycle" (Szczepanski & Trott, 1975) provides an alternative, if speculative interpretation of both our own and McNally's results, assuming that $RIB_5C$ and H-4-II-E contain a fraction of quiescent, radioresistant cells. This is a reasonable assumption for $RIB_5C$, since McNally reports a 10% hypoxic fraction and, as Tannock (1968, 1972) suggested, and Hermans & Barendsen (1978) demonstrated, these cells tend to be both radioresistant and quiescent. The existence of a quiescent cell population in H-4-II-E has not been confirmed, although all tumours examined contained cords and, as already stated, mitotic cells were more numerous in the perivascular tissue and decreased in concentration with increasing distance from the vein. On these assumptions, the proportion of quiescent and proliferative cells remaining to repopulate the tumour after irradiation will vary with dose. At low doses repopulation will be effected by both proliferative and quiescent cells and may be initiated immediately after a period of mitotic delay. High-dose treatment will leave only quiescent (resting) cells viable, thus repopulation will proceed only after the postulated period of proliferative delay. The curve relating growth delay and log surviving fraction would then be biphasic, as observed. In fact the point of inflection occurs at a surviving fraction of $10^{-1}$ in $RIB_5C$, below which, as McNally points out, cell survival is indeed attributable to the hypoxic fraction. Making the same assumptions, the effect of irradiation under hyperoxic or hypoxic conditions will be to reduce the selectivity of the treatment by eliminating oxygen gradients within the tumour. At all doses a fraction of proliferative cells will remain viable and capable of tumour repopulation at the expected rate. McNally's data show that the $T_d$ of the surviving population was 3·3 days for tumours of air-breathing animals and 1·5 days for tumours irradiated under abnormal oxygenation. $T_d$ for untreated controls was 2·7 days.

A proliferative delay was discounted by McNally because assays of the clonogenic fraction of $RIB_5C$ at intervals from irradiation showed that for similar cell kills under the 3 irradiation conditions (surviving fraction $= 10^{-2}$), all returned to a clonogenic fraction of 1 at the same time. However, the surviving fraction tells us only when dead (non-clonogenic) cell clearance is completed, not when surviving cell proliferation begins.

The time of repopulation after irradiation may of course be determined by factors other than a proliferative delay; *e.g.* the degree of cell clearance or reoxygenation. In a C3H mouse mammary tumour, changes in tumour cord radii and cellularity indicated an initial cell clearance during mitotic delay, then proliferation of sterilized cells, followed by clearance of those cells to produce a minimum of cellularity (and presumably increased nutrient and oxygen supply) before regrowth begins (Tannock & Howes, 1973). It is quite possible that reoxygenation or cell clearance is a factor in the recovery of H-4-II-E. At 7 days, when tumour growth resumed, the clonogenic fraction was a little less than 40%. This is about the same size as the clonogenic fraction (20%) in the R-1 sarcoma at the beginning of recovery after X-rays or neutrons (Barendsen & Broerse, 1969). However, such an explanation cannot by itself account for the biphasic shape of the curve relating cell survival and growth delay.

Vascular damage was the suggested cause of a chronic reduction in growth rate in the mouse adenocarcinoma C3HBA (Nelson *et al.*, 1976) and of secondary growth delay in the R-1 sarcoma (Tenforde *et al.*, 1979) and the fibrosarcoma RIB5 (Thomlinson & Craddock, 1967). Secondary growth delay is a period of slowed tumour growth that occurs after the initial period of recovery and at a volume similar to that at the time of treatment.

The observations presented and cited here have several implications: (a) They demand a precise definition of the term "repopulation". It is used here in reference to the proliferation of cells surviving irradiation. It does not refer to changes in the proportions of clonogenic to non-clonogenic cells, which may merely be a function of dead-cell clearance and not a result of clonogenic-cell proliferation. Assays of the clonogenic fraction alone are therefore inadequate to demonstrate the time of repopulation. (b) Since growth delay cannot be directly related to log cell kill and because tumour cure is necessarily dependent upon cell killing, growth delay may be a misleading measure of therapeutic effect; assuming of course that the object of therapy is cure and not remission. (c) If the interpretation of tumour cord dynamics is correct (Tannock, 1968) a proliferative delay as seen here may reduce cell loss at the cord periphery and perhaps from the tumour as a whole, in which case the interpretation of tumour volume changes after irradiation may sometimes require revision. (d) The faster growth rates in irradiated tumours than in control tumours 10–16 days after treatment offer the possibility of increasing the effectiveness of sequential therapy by giving radiation or proliferation-dependent chemotherapy agents during this period of accelerated tumour growth.

We are grateful for the skilled technical assistance given us by Martha S. MacLeod, William R. Huckle, and Shirley T. Mays.

This work was supported in part by U.S. Public Health Service Research Emphasis Grant (CREG) CA20516 for Experimental Combined Modality (Radiotherapy–Chemotherapy) Studies (ECMRC) from the National Cancer Institute.

### REFERENCES

BARENDSEN, G. W. & BROERSE, J. J. (1969) Experimental radiotherapy of a rat rhabdomyosarcoma with 15 MeV neutrons and 300kV X-rays: I: Effects of single exposures. *Eur. J. Cancer*, **5**, 373.

BURTON, K. (1956) A study of the conditions and mechanism of diphenylamine reaction for the colorimetric estimation of deoxyribonucleic acid. *Biochem. J.*, **62**, 315.

DENEKAMP, J. & HARRIS, S. R. (1976) Studies of the processes occurring between two fractions in experimental mouse tumors. *Int. J. Radiat. Oncol. Biol. Phys.*, **1**, 421.

EVANS, M. J. & KOVACS, C. J. (1977) Properties of the H-4-II-E tumor cell system: I: Growth and cell proliferation kinetics of an experimental hepatoma. *Cell Tiss. Kinet.*, **10**, 233.

HERMENS, A. F. (1973) Variations in the cell kinetics and growth rate in an experimental tumour during natural growth and after irradiation. *Thesis, University of Amsterdam.*

HERMENS, A. F. & BARENDSEN, G. W. (1978) The proliferative status and clonogenic capacity of tumour cells in a transplantable rhabdomyosarcoma of the rat before and after irradiation with 800 rad of X-rays. *Cell Tiss. Kinet.*, **11**, 83.

HOPKINS, H. A., KOVACS, C. J., LOONEY, W. B. & WAKEFIELD, J. A. (1976) Differential recovery of intestine, bone marrow and thymus following 5-fluorouracil administration. *Cancer Biochem. Biophys.*, **1**, 303.

JANIK, P. & STEEL, G. G. (1972) Cell proliferation during immunological perturbation in three transplanted tumours. *Br. J. Cancer*, **26**, 108.

KOVACS, C. J., EVANS, M. J. & HOPKINS, H. A. (1977) Properties of the H-4-II-E tumor cell system: II: *In vitro* characteristics of an experimental tumor cell line. *Cell Tiss. Kinet.*, **10**, 245.

LOONEY, W. B., HOPKINS, H. A., GROVER, W. H., MACLEOD, M. S., RITENOUR, E. R. & HOBSON, A. S. (1980*a*) Solid tumor models for the assessment of different treatment modalities: XIII: Comparison of response and recovery of host and solid tumor to cyclophosphamide and radiation. *Cancer* **45**, 2793.

LOONEY, W. B., RITENOUR, E. R. & HOPKINS, H. A. (1980*b*) Changes in growth rate of an experimental solid tumor following increasing doses of cyclophosphamide. *Cancer Research* **40**, 2179.

MCNALLY, N. J. (1973) A comparison of the effects of radiation on tumour growth delay and cell survival. The effect of oxygen. *Br. J. Radiol.*, **46**, 450.

NELSON, J. S. R., CARPENTER, R. E. & DURBORAW, D. (1976) Mechanisms underlying reduced growth rate in C3HBA mammary adenocarcinomas recurring after single doses of X-rays or fast neutrons. *Cancer Res.*, **36**, 524.

STEPHENS, T. C. & STEEL, G. G. (1980) Regeneration of tumors after cytotoxic treatment. In *Radiation Biology in Cancer Research*. Eds Meyn & Withers. New York: Raven Press. p. 385.

SZCZEPANSKI, L. V. & TROTT, K. R. (1975) Post-irradiation proliferation kinetics of a serially transplanted murine adenocarcinoma. *Br. J. Radiol.*, **48**, 200.

TANNOCK, I. (1968) The relation between cell proliferation and the vascular system in a transplanted mouse mammary tumour. *Br. J. Cancer*, **22**, 258.

TANNOCK, I. (1972) Oxygen diffusion and the distribution of cellular radiosensitivity in tumours. *Br. J. Radiol.*, **45**, 515.

TANNOCK, I. & HOWES, A. (1973) The response of viable tumor cords to a single dose of radiation. *Radiat. Res.*, **55**, 477.

TENFORDE, T. S., CURTIS, S. B., WOODRUFF, H. K. & 5 others (1979) Studies on the regrowth rate, morphological characteristics and transplantation properties of rat rhabdomyosarcoma tumours following large doses of X-rays. *Int. J. Rad. Biol.*, **35**, 589.

THOMLINSON, R. H. & CRADDOCK, E. A. (1967) The gross response of an experimental tumour to single doses of X-rays. *Br. J. Cancer*, **9**, 108.

TWENTYMAN, P. R. & BLEEHEN, N. M. (1975) Studies of "potentially lethal damage" in EMT6 mouse tumour cells treated with bleomycin either *in vitro* or *in vivo*. *Br. J. Cancer*, **32**, 491.

VAN PEPERZEEL, H. A. (1972) Effects of single doses of radiation on lung metastases in man and experimental animals. *Eur. J. Cancer*, **8**, 665.


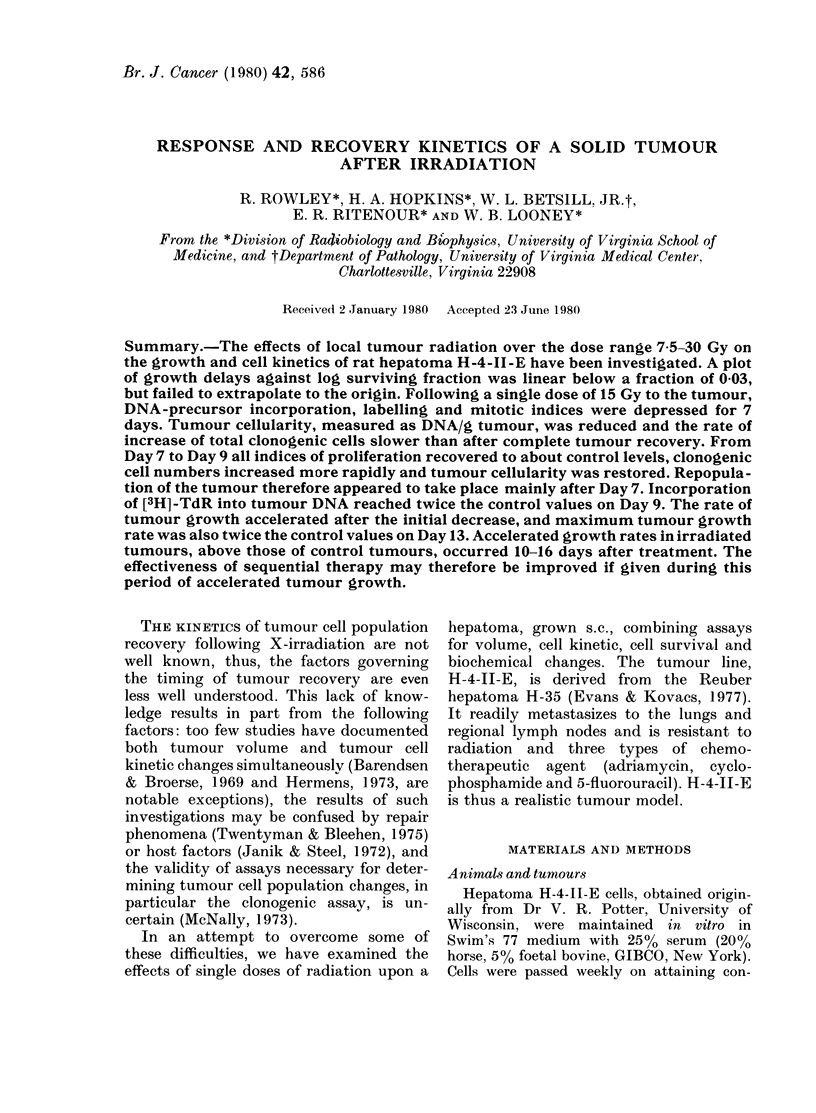

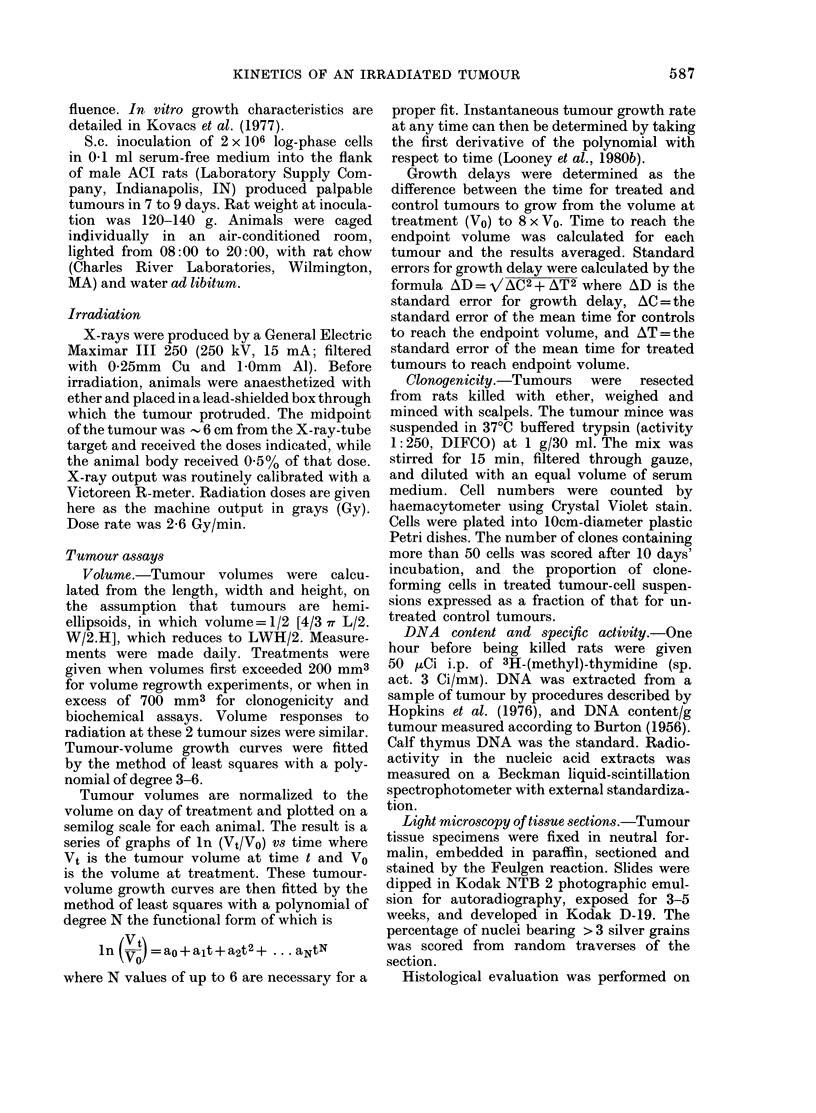

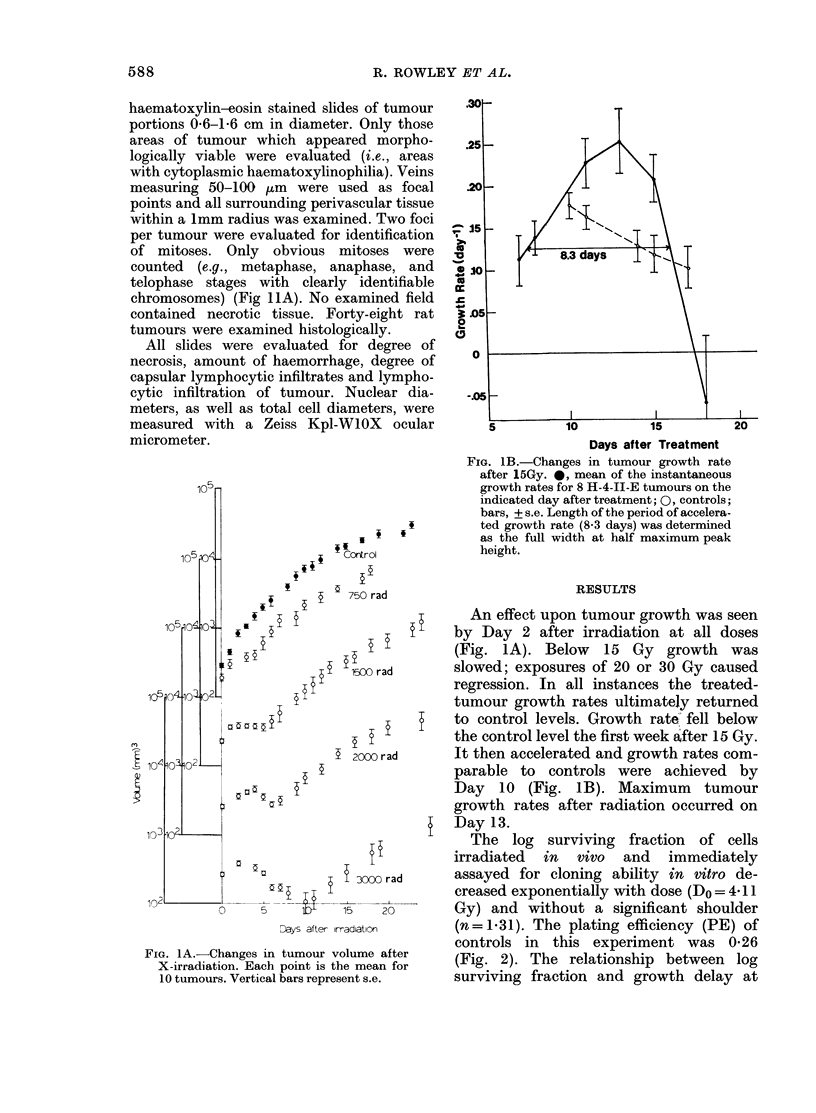

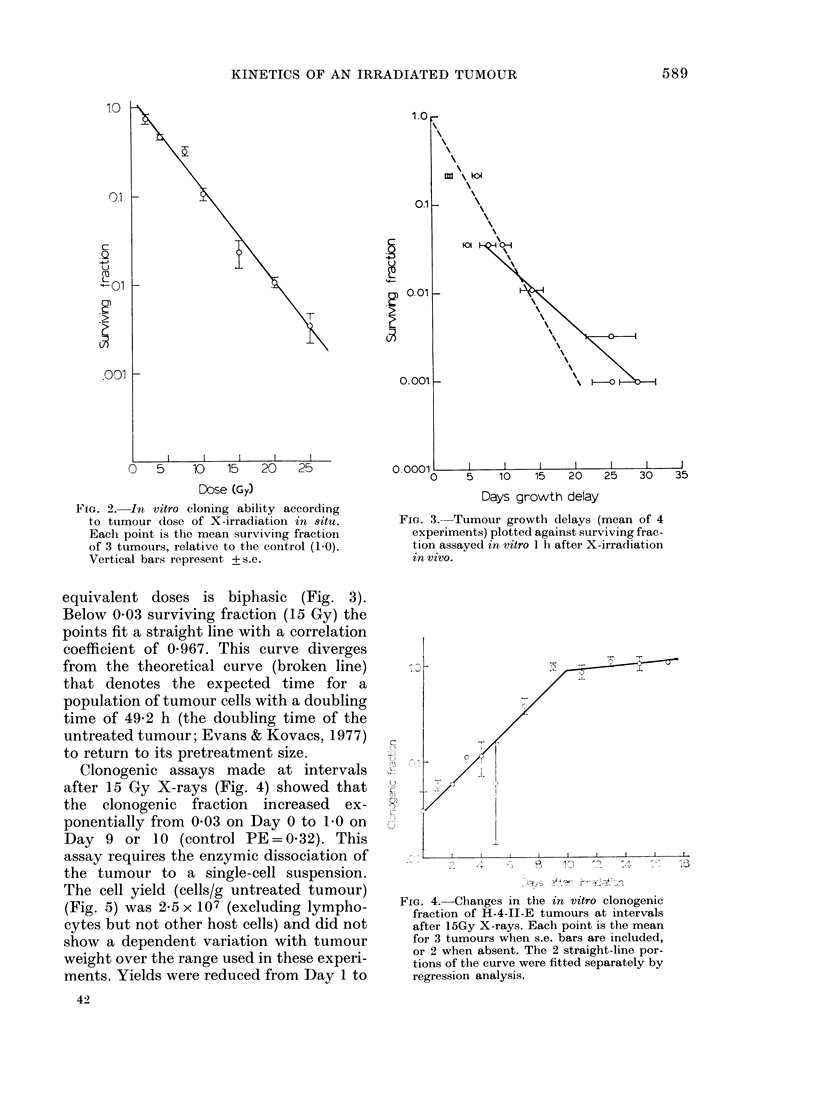

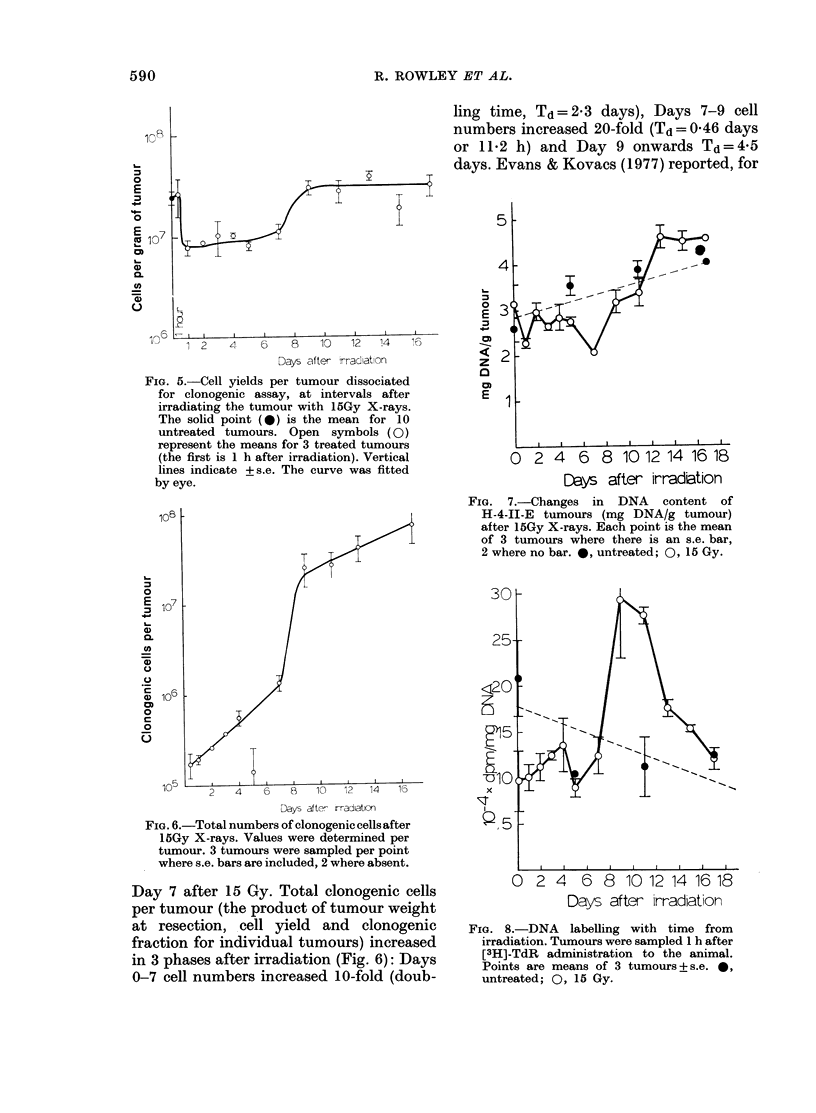

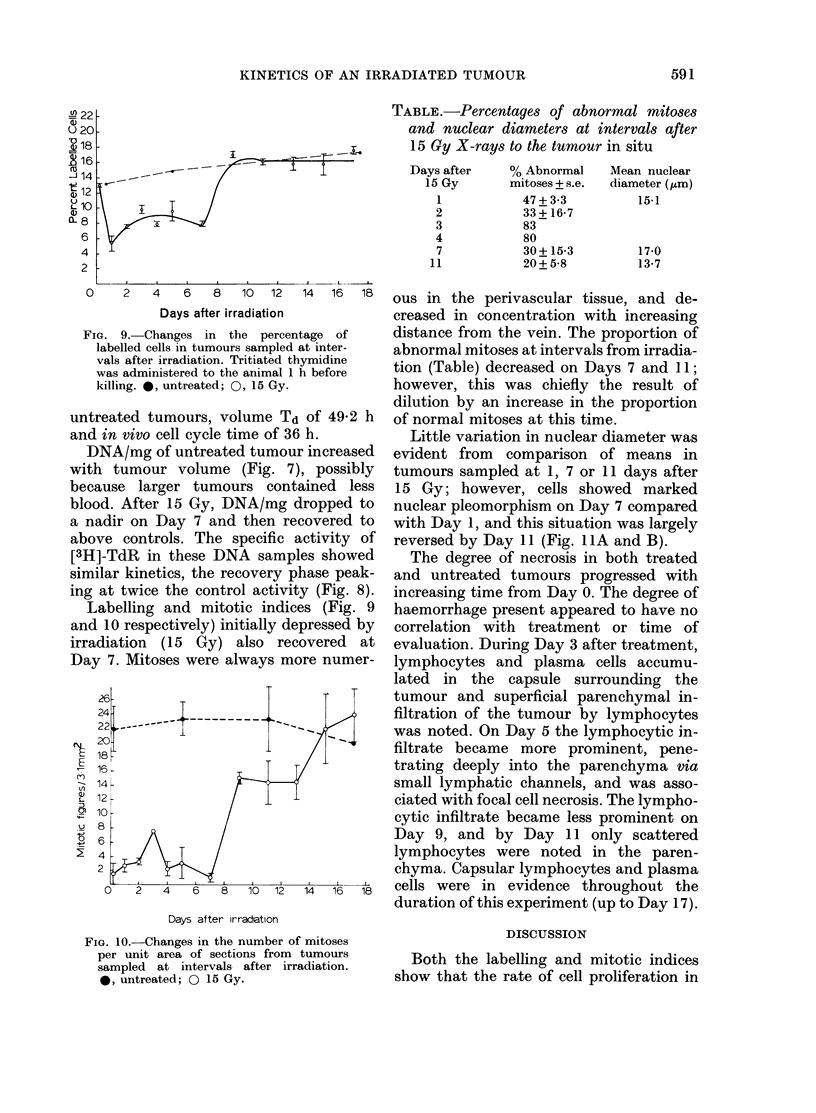

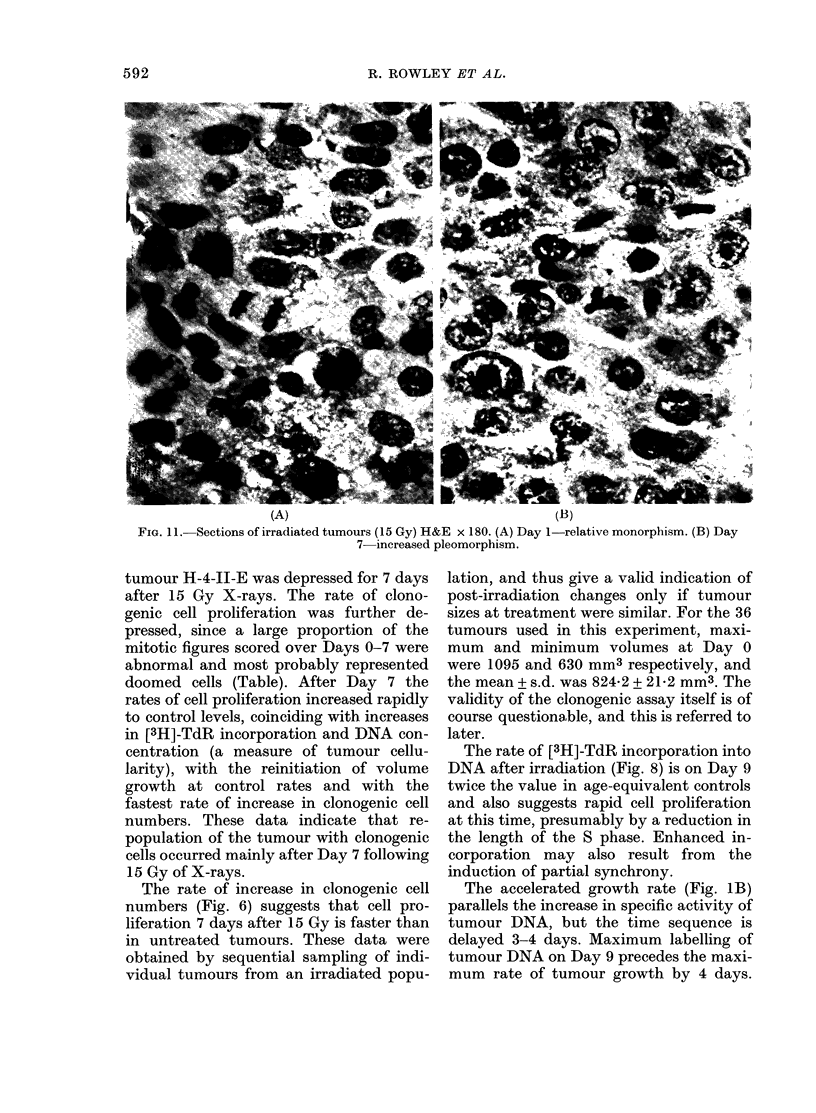

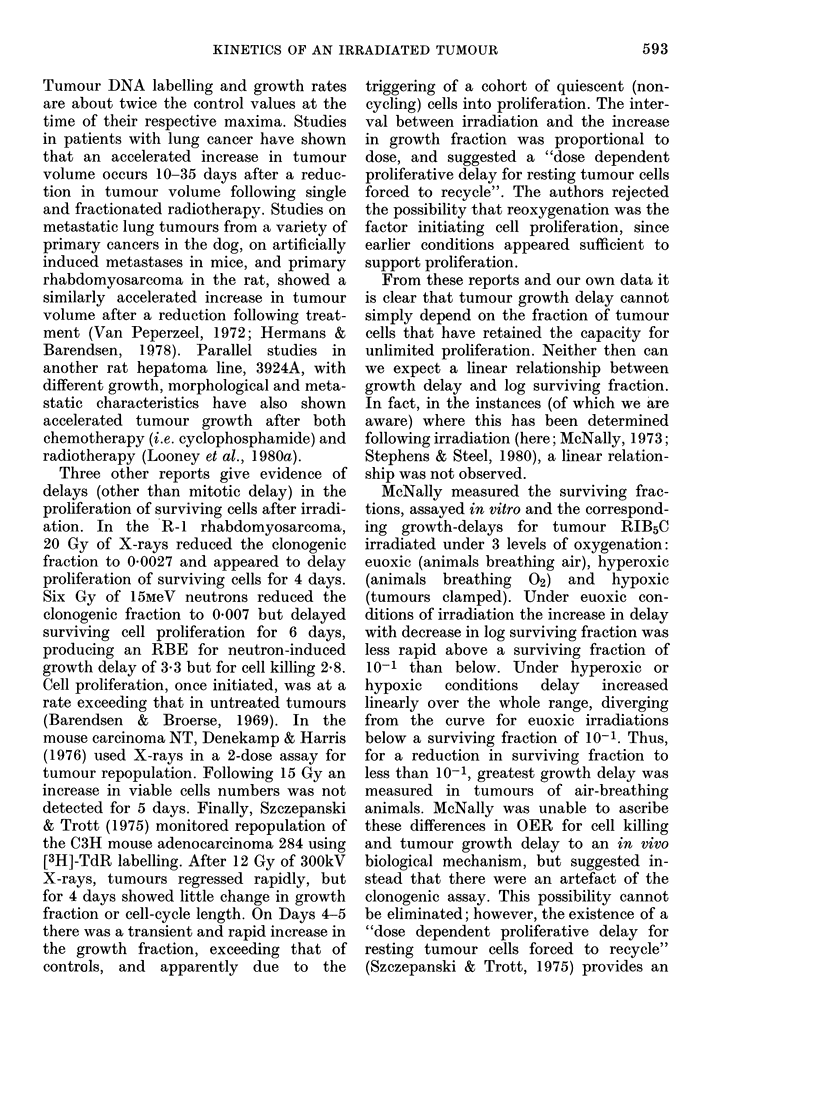

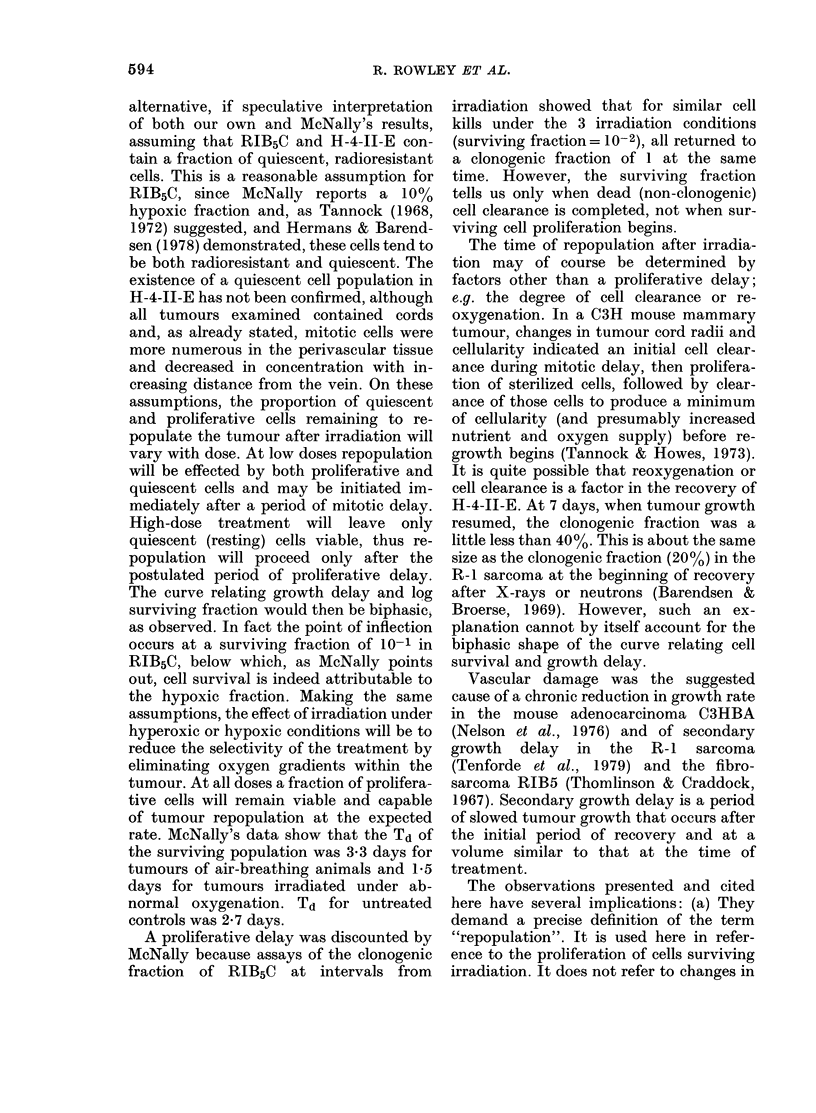

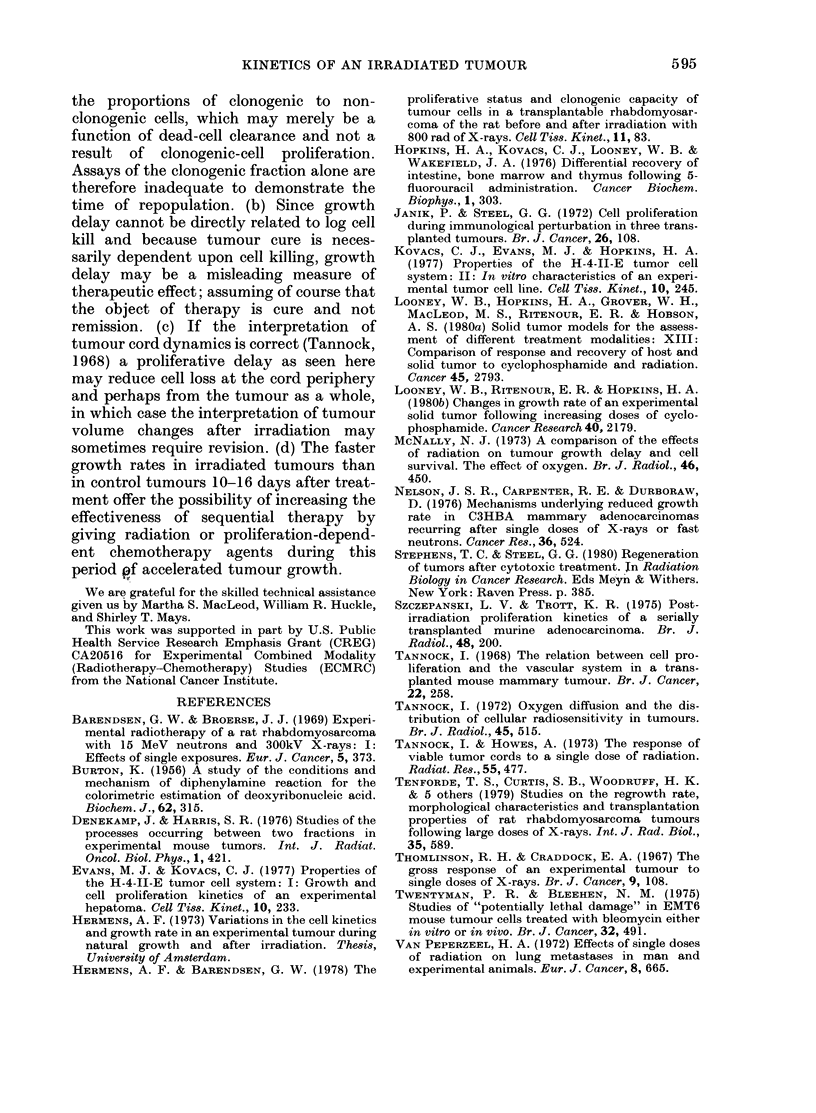


## References

[OCR_00983] BURTON K. (1956). A study of the conditions and mechanism of the diphenylamine reaction for the colorimetric estimation of deoxyribonucleic acid.. Biochem J.

[OCR_00978] Barendsen G. W., Broerse J. J. (1969). Experimental radiotherapy of a rat rhabdomyosarcoma with 15 MeV neutrons and 300 kV x-rays. I. Effects of single exposures.. Eur J Cancer.

[OCR_00989] Denekamp J., Harris S. R. (1976). Studies of the processes occurring between two fractions in experimental mouse tumors.. Int J Radiat Oncol Biol Phys.

[OCR_00995] Evans M. J., Kovacs C. J. (1977). Properties of the H-4-II-E tumor cell system. I. Growth and cell proliferation kinetics of an experimental hepatoma.. Cell Tissue Kinet.

[OCR_01007] Hermens A. F., Barendsen G. W. (1978). The proliferative status and clonogenic capacity of tumour cells in a transplantable rhabdomyosarcoma of the rat before and after irradiation with 800 rad of X-rays.. Cell Tissue Kinet.

[OCR_01014] Hopkins H. A., Kovacs C. J., Looney W. B., Wakefield J. A. (1976). Differential recovery of intestine, bone marrow, and thymus of rats with solid tumors following 5-fluorouracil administration.. Cancer Biochem Biophys.

[OCR_01021] Janik P., Steel G. G. (1972). Cell proliferation during immunological perturbation in three transplanted tumours.. Br J Cancer.

[OCR_01026] Kovacs C. J., Evans M. J., Hopkins H. A. (1977). Properties of the H-4-II-E tumor cell system. II. In vitro characteristics of an experimental tumor cell line.. Cell Tissue Kinet.

[OCR_01031] Looney W. B., Hopkins H. A., Grover W. H., Macleod M. S., Ritenour E. R., Hobson A. S. (1980). Solid tumor models for the assessment of different treatment modalities. XIII. Comparison of response and recovery of host and solid tumor to cyclophosphamide and radiation.. Cancer.

[OCR_01040] Looney W. B., Ritenour E. R., Hopkins H. A. (1980). Changes in growth rate of an experimental solid tumor following increasing doses of cyclophosphamide.. Cancer Res.

[OCR_01046] McNally N. J. (1973). A comparison of the effects of radiation on tumour growth delay and cell survival. The effect of oxygen.. Br J Radiol.

[OCR_01052] Nelson J. S., Carpenter R. E., Durboraw D. (1976). Mechanisms underlying reduced growth rate in C3HBA mammary adenocarcinomas recurring after single doses of x-rays or fast neutrons.. Cancer Res.

[OCR_01065] Szczepanski L. v., Trott K-R (1975). Post-irradiation proliferation kinetics of a serially transplanted murine adenocarcinoma.. Br J Radiol.

[OCR_01077] Tannock I. F. (1972). Oxygen diffusion and the distribution of cellular radiosensitivity in tumours.. Br J Radiol.

[OCR_01071] Tannock I. F. (1968). The relation between cell proliferation and the vascular system in a transplanted mouse mammary tumour.. Br J Cancer.

[OCR_01082] Tannock I., Howes A. (1973). The response of viable tumor cords to a single dose of radiation.. Radiat Res.

[OCR_01087] Tenforde T. S., Curtis S. B., Woodruff H. K., Parks D. L., Daniels S. J., Crabtree K. E., Schilling W. A., DeGuzman R. J. (1979). Studies on the regrowth rate, morphological characteristics and transplantation properties of rat rhabdomyosarcoma tumours following large doses of X-rays.. Int J Radiat Biol Relat Stud Phys Chem Med.

[OCR_01095] Thomlinson R. H., Craddock E. A. (1967). The gross response of an experimental tumour to single doses of x-rays.. Br J Cancer.

[OCR_01100] Twentyman P. R., Bleehen N. M. (1975). Studies of "potentially lethal damage" in EMT6 mouse tumour cells treated with bleomycin either in vitro or in vivo.. Br J Cancer.

[OCR_01106] van Peperzeel H. A. (1972). Effects of single doses of radiation on lung metastases in man and experimental animals.. Eur J Cancer.

